# Prism adaptation aftereffects in stroke patients with spatial neglect: Pathological effects on subjective straight ahead but not visual open-loop pointing

**DOI:** 10.1016/j.neuropsychologia.2007.11.005

**Published:** 2008

**Authors:** Margarita Sarri, Richard Greenwood, Lalit Kalra, Ben Papps, Masud Husain, Jon Driver

**Affiliations:** a UCL Institute of Cognitive Neuroscience, University College London, 17 Queen Square, London, WC1N 3AR, UK; b Regional Neurological Rehabilitation Unit, Homerton University Hospital, London, UK; c Department of Diabetes, Endocrinology and Internal Medicine, Guy's, King's and St Thomas’ School of Medicine, Denmark Hill Campus, London, UK

**Keywords:** Neglect, Prism adaptation, Straight ahead pointing, Cancellation, Rehabilitation, Lesion anatomy

## Abstract

Prism adaptation to rightward optical shifts during visually guided pointing is considered a promising intervention in right-hemisphere stroke patients with left spatial neglect. Conventionally, prism adaptation is assessed via *aftereffects*, on subjective straight ahead (SSA) pointing with eyes closed; or by visual open-loop pointing (VOL), i.e. pointing to a visual target without seeing the hand. Previous data suggest indirectly that prism aftereffects in neglect patients may be larger (pathologically so) when assessed by SSA than by VOL. But these measures have never been directly compared within the same patients after identical prism exposure. Accordingly we implemented both measures here within the same group of 13 neglect patients and 13 controls. Prism aftereffects were much larger for SSA than VOL in neglect patients, falling outside the normative range only for SSA. This may arise because the SSA task can itself involve aspects of neglect that may be ameliorated by the prism intervention, hence showing abnormal changes after prisms. The extent of SSA change after prisms varied between patients, and correlated with improvements on a standard cancellation measure for neglect. The lesions of patients who did versus did not show neglect improvement immediately after prisms provide an initial indication that lack of improvement may potentially relate to cortical damage in right intraparietal sulcus and white matter damage in inferior parietal lobe and middle frontal gyrus. Future studies of possible rehabilitative impact from prisms upon neglect may need to consider carefully how to measure prism adaptation per se, separately from any impact of such adaptation upon manifestations of neglect.

## 1 Introduction

Spatial neglect is a common and disabling syndrome after major stroke that poses a significant challenge for neurological rehabilitation and is a negative prognostic factor for functional outcome (e.g. Coulthard, Singh-Curry, & Husain, 2006; Luaute, Halligan, Rode, Rossetti, & Boisson, 2006). Spatial neglect is now recognised to be a multi-component syndrome (e.g. see Bartolomeo, Thiebaut de Schotten, & Doricchi, 2007; Danckert & Ferber, 2006; Driver, Vuilleumier, & Husain, 2004; Husain & Nachev, 2007; Karnath, Milner, & Vallar, 2002; Mesulam, 1999; Milner & McIntosh, 2005). The observed symptoms typically include losses of awareness and exploration towards the contralesional side of space, even when primary sensory and motor functions are intact. Neglect patients may fail to acknowledge contralesional stimuli, and may even neglect contralesional parts of their own body or of mental representations. When exploring a scene, their eye, head, body and hand-movements may fail to be directed towards leftward elements (e.g. Karnath, Niemeier, & Dichgans, 1998; Marotta, McKeeff, & Behrmann, 2003) despite an ability to make such exploratory movements when prompted.

Prism adaptation therapy has recently been identified as a promising rehabilitative intervention in neglect, with numerous studies following on from the pioneering study by Rossetti et al. (1998). A short period of prism adaptation, caused by pointing to visual targets while wearing prisms that induce a rightward optical shift, has now been found to ameliorate numerous aspects of the neglect syndrome, in both acute and chronic patients, on a variety of measures including conventional visuo-manual clinical measures (e.g. cancellation; line bisection) and various other tasks (e.g. tactile extinction; reading; mental imagery; ocular and haptic exploration), with improvements reported that may last several hours, days or perhaps even weeks (see e.g. Angeli, Benassi, & Ladavas, 2004; Farne, Rossetti, Toniolo, & Ladavas, 2002; Maravita et al., 2003; McIntosh, Rossetti, & Milner, 2002; Pisella, Rode, Farne, Boisson, & Rossetti, 2002; Rode et al., 2006; Rossetti et al., 1998, 2004; Sarri, Kalra, Greenwood, & Driver, 2006; Serino, Angeli, Frassinetti, & Ladavas, 2006; Striemer & Danckert, 2007; Tilikete et al., 2001). However not all findings have been uniformly positive, nor have all measures always been affected (e.g. see Dijkerman et al., 2003; Ferber, Danckert, Joanisse, Goltz, & Goodale, 2003; Morris et al., 2004; Rousseaux, Bernati, Saj, & Kozlowski, 2006; Sarri et al., 2006).

The typical procedure in prism interventions comprises a short adaptation period, during which neglect patients point to visual targets while wearing prisms that induce a rightward optical shift of ∼10–15°. This initially leads to errors of pointing to the right of the visual target, leading in turn to compensatory leftward manual corrections. In normals and patients, this compensatory behaviour is typically followed by an ‘aftereffect’ when the prisms are removed (cf. Held & Gottlieb, 1958), with manual errors now being biased towards the left instead. The adaptive aftereffect is thought to involve compensatory shifts of proprioceptive and/or visuo-motor representations following exposure to prisms (in addition to any strategic factors, see Section 4). The observed aftereffect is traditionally considered as the critical dependent-measure showing that some form of adaptation has taken place (see e.g. Redding & Wallace, 1993; Welch, 1978).

Two different measures are widely used to measure such prism aftereffects, in both the normal and the neuropsychological literature. These comprise so-called ‘proprioceptive’ pointing to the subjective straight ahead (SSA), with eyes closed or blindfolded; or visual open-loop pointing (VOL) to visual targets with the responding hand unseen to preclude visual feedback about hand position. These aftereffect measures are often considered interchangeably, but a few normal studies have compared them directly (e.g. Beckett & Melamed, 1980; Guan & Wade, 2000; Redding & Wallace, 1993, 2000, 2001). Recent theoretical analyses for normals have emphasized that there may be important differences between the two measures, most obviously in the role for vision in providing objective target information in VOL, versus a strictly subjective midline requirement in SSA (see Hatada, Rossetti, & Miall, 2006).

Here we emphasize that it may be especially important to distinguish these two different measures of prism aftereffects when dealing with neglect patients. In the pioneering study of prism effects in neglect, Rossetti et al. (1998) found that patients with left neglect demonstrated prism aftereffects on SSA that were pathologically large following a rightward optical prismatic shift. Based on these and subsequent results (Ferber et al., 2003; Pisella et al., 2002; Rode, Rossetti, & Boisson, 2001; Rode, Rossetti, Li, & Boisson, 1998), it has been argued that neglect patients may show unusually large prism aftereffects (e.g. see Redding & Wallace, 2006; Rossetti et al., 1998; Rossetti & Rode, 2002). But here we emphasize that since a distorted SSA (usually with a pathological bias to the right) is often considered to provide one key aspect of the left neglect syndrome (see e.g. Jeannerod & Biguer, 1987; Karnath, 1994, 1997), then prism effects upon this particular SSA measure might be considered as one manifestation of beneficial prism impacts upon components of the neglect syndrome, rather than as providing a strictly ‘neglect-free’ measure of prism adaptation per se. For instance, Karnath (1994, 1997) has argued that a rightward shift in the SSA may be a fundamental aspect of neglect that might explain or contribute to several of its other manifestations.

Several prior studies have indeed indicated that neglect patients often show substantial pathological rightward biases in SSA tasks (e.g. Chokron & Bartolomeo, 2000; Ferber & Karnath, 1999; Heilman, Bowers, & Watson, 1983; Jeannerod & Biguer, 1987; Karnath & Fetter, 1995; Richard, Honore, Bernati, & Rousseaux, 2004). By contrast, many neglect patients appear to be reasonably accurate when pointing to a visual target without feedback in open loop (e.g. Angeli, Benassi, et al., 2004; Coulthard, Parton, & Husain, 2006; Farne et al., 2002; Frassinetti, Angeli, Meneghello, Avanzi, & Ladavas, 2002; Jackson, Newport, Husain, Harvey, & Hindle, 2000; Maravita et al., 2003). Given that prism therapy has now been shown to affect many aspects of the neglect syndrome, it might in principle also have an impact on distortions of the SSA in neglect patients, over and above any prism adaptation aftereffect per se that arises, as might be measured more purely with the VOL task (see also Perenin, 1997; Pisella et al., 2002). If so, then we would expect neglect patients to show a bigger impact from prism therapy on the SSA than on the VOL measure. We might also expect that the impact on the SSA in particular may relate to any more general beneficial impact on neglect, as assessed with other standard clinical measures (e.g. cancellation).

When considering the extant literature on prism therapy in neglect with this point in mind, there is some indirect suggestive evidence that those studies reporting unusually large prism aftereffects in neglect patients, after exposure to rightward optical deviation, are typically those employing the SSA measure (Ferber et al., 2003; Pisella et al., 2002; Rode et al., 1998, 2001, 2006; Rossetti et al., 1998). By contrast, those studies using a visual open-loop (VOL) measure of prism aftereffects appear to have observed somewhat smaller effects in neglect patients (Angeli, Benassi, et al., 2004; Angeli, Meneghello, Mattioli, & Ladavas, 2004; Farne et al., 2002; Frassinetti et al., 2002; Maravita et al., 2003; Rossetti et al., 2004; Sarri et al., 2006). Here we implemented a meta-analysis of these previously published studies, all using 10° prisms and focusing on patients diagnosed with spatial neglect (see also review by Redding & Wallace, (2006)). Our new meta-analysis indicates that prism aftereffects in neglect patients were on average more than double in size for those studies employing an SSA measure (grand mean of reported mean aftereffects was 10.18°, S.D. = 3.50°) than for those using a VOL measure (grand mean 4.15°, S.D. = 1.5°), a significant difference (*t*(11) = 4.15, *p* *=* 0.002).

Thus, prism aftereffects as measured by SSA in neglect patients, following exposure to rightward optical shifts, appear pathologically large when compared to those measured by VOL. They also seem pathologically large, for the SSA measure in particular, in comparison to normal controls under similar conditions (e.g. see Rossetti et al., 1998), as addressed further below. But to our knowledge, SSA and VOL prism aftereffects have never been compared directly *within* the same group of neglect patients as yet, after an identical prism intervention. It is thus uncertain whether the apparent differences between SSA and VOL outcomes in neglect patients (as implied by our meta-analysis) genuinely reflect the nature of the task used to index prism aftereffects, or instead some differences between individual patients or the slightly different protocols used in different studies.

Accordingly, here we implemented both SSA and VOL measures of prism aftereffects in a group of 13 consecutive patients with varying severity of clinical neglect, to determine within the same patients, after exactly the same prism protocol, whether SSA aftereffects would indeed be significantly larger than VOL aftereffects. Moreover, for comparison we also implemented both SSA and VOL measures of prism aftereffects in an age-matched healthy control group. For the reasons given above a larger effect on the SSA measure than the VOL was predicted (pathologically so) for the patients, but not for the healthy controls.

## 2 Methods

### 2.1 Participants

A series of 13 consecutive right-hemisphere stroke patients with left neglect (six females, seven males, mean age 57.07, S.D. = 14.17) were recruited. All gave informed consent to participate in accord with local ethics, and showed left neglect on some standard clinical measures, as described below. MRI or CT scans revealed unilateral right lesions due to stroke in all cases. On clinical examination, all patients were alert and well oriented in time and space. Patients were selected on the basis of impaired performance on several standard tests for visuo-spatial neglect, including the Mesulam shape cancellation test (Weintraub & Mesulam, 1985); line bisection; identification of the left side of chimeric figures; emotional judgements on chimeric face stimuli (e.g. Mattingley, Bradshaw, Phillips, & Bradshaw, 1993); copying of drawings; reading of single words or non-words. Diagnosis of left visual neglect was based on the presence of at least two of the following six criteria: at least 30% omissions on the left side of the page for the cancellation test; minimum rightward deviation of 12% or more in the line bisection task; minimum of 30% omissions on the left side of chimeric object figures during identification; selection of the right-smiling face as ‘happier’ in at least 75% of the chimeras presented; omission of left sided elements in the drawing test; omission or misidentification of left sided elements in the reading test. Five out of 13 patients (KP, EH, PH, LG and MM) also had complete left homonymous hemianopia on confrontation. Details of age, gender, time post-injury, lesion site and pathology, together with scores on standard neglect tests, are given in Table 1.

Thirteen right-handed, age-matched healthy participants (eight females, five males; mean age: 53 years, S.D. = 9.86), with normal or corrected vision were tested as control subjects.

### 2.2 Design and procedure

Patients and controls underwent the same experimental procedure. Each sat at a table in front of the experimenter. Prism aftereffects were assessed by means of two open-loop pointing tasks: subjective straight ahead (SSA) pointing with blindfold; visual open-loop pointing (VOL). Measures in the two pointing tasks (SSA and VOL) were obtained immediately before and immediately after the prism adaptation procedure. The order of presentation for the two pointing tasks (i.e. SSA and VOL) was counterbalanced between subjects but held constant before and after prism adaptation for each subject. For the patients only, the Mesulam shape cancellation task (Weintraub & Mesulam, 1985) was also used to assess spatial-exploration performance before and after prisms. This cancellation task was always given after both pointing tasks had been completed, both pre- and post-prism exposure. The experimenter ensured that the subject's head and trunk were kept straight during testing.

#### 2.2.1 Subjective straight ahead pointing (SSA)

For the SSA task, subjects were blindfolded and asked to make ten free pointing movements straight ahead; i.e. in alignment with their perceived mid-sagittal axis, using their right arm, starting each pointing movement at the command of the experimenter. They were instructed to fully extend their arm, placing their right index finger on the table in front of them, in line with their perceived mid-sagittal axis, and to leave it there until the experimenter gave the verbal command to prepare for the next pointing. Subjects were instructed to start by placing their hand always at the same position on their sternum and to return their hand there after every pointing movement. Subjects made 10 such blindfolded SSA pointing responses prior to the prism manipulation, and then a further 10 afterwards, to provide pre-/post-measures. Pointing endpoints were marked by the examiner on a tape attached to the table (unseen by the subjects at any point in the experiment), thus allowing recording of the pointing position in relation to the objective body midline (as defined by the subjects’ mid-sagittal axis, as also recorded by the experimenter). The pointing error was calculated as the visual angle between the recorded pointing position and the objective body midline, from the patient's perspective, with negative numbers indicating leftward error and positive numbers rightward error.

#### 2.2.2 Visual open-loop pointing (VOL)

To obtain a measure of visual open-loop (VOL) target pointing, subjects were asked to make repeated pointing movements to a single visible target placed at the actual centre of their mid-sagittal plane (so that the correct response was identical to the objectively correct response for the SSA task, see above). Each subject made 10 VOL responses with his/her right arm before the prism adaptation procedure, plus 10 after removing the prisms, to provide pre-/post-measures. Vision of the hand was obscured throughout this aspect of the procedure by means of an occluding cardboard panel (65 cm × 58 cm) which was fixed at a level just below the subject's chin. This panel completely occluded the subject's view of their hands during both rest and pointing movements. The visual target was a red dot fixed at the distal end of the panel, aligned with the subject's mid-sagittal plane (at a distance of ∼55 cm). Subjects were instructed to make fast movements under the panel (i.e. with no visual feedback), to indicate the position of the visual target at the command of the experimenter, pointing with their index finger, and then returning their arm to the initial starting position on their chest at the level of their sternum, after the experimenter had marked the terminal pointing position on the vertical section of the panel (unseen by the subjects). Again for this task negative numbers indicate leftward error and positive numbers rightward error, in degrees of visual angle from the patient's perspective.

Note that the SSA and VOL tasks were thus analogous in several respects. Ten pre-and 10 post-measures were taken for each, within a short space of time (roughly a minute each). Moreover both tasks required pointing to a single central location, and the objective (correct) response required for each was identical.

#### 2.2.3 Prism adaptation procedure

During the prism adaptation procedure, the subjects wore base-left wedge prisms that induced a 10° optical shift to the right (Société Optique Peter, Lyon; as also used by Rossetti et al., 1998). Exposure to the prismatic shift was accomplished by having subjects perform 80 repeated pointings with their right hand to two visual targets placed on a table, 10° to the left or right (40 trials each) of the centre of their mid-sagittal plane, at a distance of ∼55 cm from their trunk, in a randomly intermingled order. Subjects were instructed to make fast movements to the unpredictable left or right target location, and then return their arm to the initial starting position on the table by their trunk centre. The initial position of their arm was occluded by a horizontal board, occluding approximately 25% of the distance between the patient and the targets (see also e.g. Rossetti et al., 1998). Hence subjects could see their arm only while moving it towards the target, with closed-loop visual feedback for any terminal errors, thus inducing corrections and adaptation to the prismatic deviation. Total exposure to the prisms was approximately 10 min for each patient, and prisms were then removed prior to retesting of the SSA, VOL and cancellation measures.

## 3 Results

Although the pointing error reduction during prism exposure was not formally measured, all 13 patients (and healthy controls) appeared to adapt well to the prismatic optical displacement induced during exposure, with errors reducing and the experimenter judging that each patient made no remaining errors in pointing before the prisms were removed after the 80 exposure trials. Moreover all patients (and controls) showed the expected leftward direction of aftereffect shift for both SSA and VOL tasks, as measured immediately after exposure to prisms, indicating that the adaptation procedure was successful for all.

### 3.1 Prism effects on subjective straight ahead (SSA) and visual open-loop pointing (VOL)

For our patient group, the mean pointing error prior to the adaptation procedure was larger in SSA than in VOL. On a case-by-case basis, all patients showed individually a rightward bias in their judgment of straight ahead pointing (SSA) in the pre-prism phase, ranging from 4.41° to a large 27.09° rightward error (mean = 8.76° rightward, S.D. = 5.97°). In the VOL task all patients except LG showed a small initial pre-prism deviation error towards the right when pointing to a fixed centrally located target (mean 1.81°; S.D. = 2.44°). This was significantly smaller than the larger mean pre-prism error of 8.76° rightward in the SSA task (*t*(12) = 4.0, *p* = 0.002). Thus, as a group the neglect patients showed a substantial pre-exposure rightward deviation in the SSA task, but less so in the VOL task, as was expected.

**Fig. 1:**
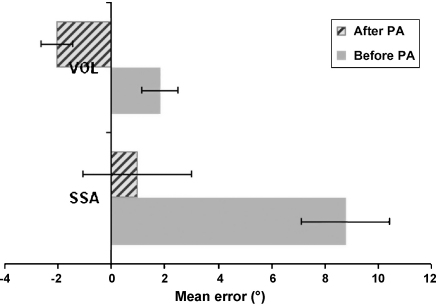
Mean pointing deviation error across all 13 patients before and after the prism adaptation procedure (PA), for the visual open-loop pointing task (VOL) and the subjective straight ahead pointing (SSA) task, showing a leftward post-prism shift in both tasks. Numbers on the *x*-axis represent pointing error in degrees (from the patient's perspective), with positive values indicating deviation to the right and negative values indicating deviation to the left. Note the strong SSA pre-prism error towards the right, and the large difference in the impact of prism adaptation on SSA vs. VOL, with the prism-induced leftward shift being much larger for SSA.

**Fig. 2:**
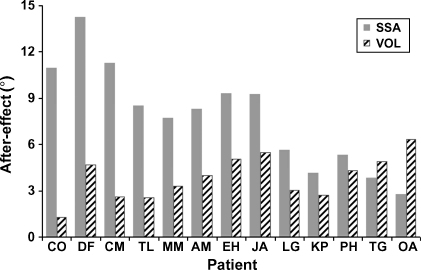
Size of aftereffect (post- minus pre-prism pointing error) for each individual patient in visual open-loop (VOL) and subjective straight ahead pointing (SSA) tasks. Numbers on the *y*-axis represent leftward shift in degrees of visual angle. Individual patients are arranged along the *x*-axis in terms of showing a larger improvement in the SSA task as compared to the VOL task.

More importantly, after the prism adaptation procedure the mean pointing error shifted significantly towards the left for both tasks. But this prism-induced shift was much more substantial for the SSA task than the VOL task in the patients (see Fig. 1). A significant leftward shift in SSA pointing (relative to the pre-prism measure) was observed for all patients (*p* < 0.005 in every case), leading to an average post-prism response that was now very close to the objective body midline (mean = 0.97° to the right, S.D. = 7.38°). A significant leftward shift in VOL was also observed for all 13 patients post-exposure (*p* < 0.05 in all cases), leading to an average post-prism response that was now −2.05° (S.D. = 2.11°) towards the left. See Fig. 1 for patient group results and Fig. 2 for individual patient results; note that the SSA aftereffect is larger than the VOL aftereffect in 11 out of 13 cases.

At a group level, applying a 2 × 2 repeated measures ANOVA on the patients’ data revealed both a significant main effect of task (SSA versus VOL; *F*(1,12) = 7.8, *p* = 0.016), with larger rightward error for SSA overall; and a significant main effect of session (pre- versus post-prism adaptation; *F*(1,12) = 166.5, *p* < 0.0001), with larger rightward error prior to prisms overall. Critically, a significant interaction between task and pre-/post-session was found *F*(1,12) = 12.7, *p* = 0.004), with the post- versus pre-shift being significantly larger for SSA (mean shift = 7.79°, S.D. = 3.34°) than for VOL (mean shift = 3.86°, S.D. = 1.42°) in the patients. Thus across the patient group, the effect of prism adaptation on SSA in our neglect patients was significantly larger than that on VOL, as we had predicted. Furthermore, the size of prism-induced shifts on SSA and VOL appears unrelated, as no correlation was found between these (*ρ*(11) = −0.286, *p* = 0.344).

**Fig. 3:**
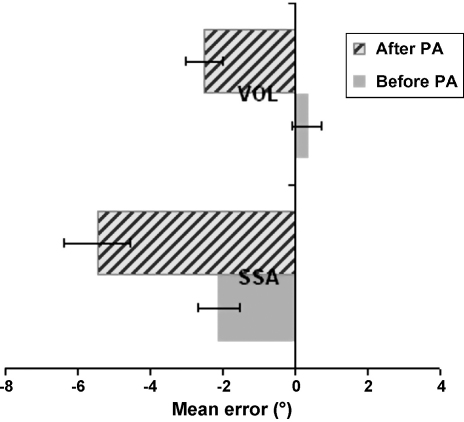
Mean pointing deviation error across all 13 healthy controls before and after the prism adaptation procedure (PA) for the visual open-loop pointing task (VOL) and for the subjective straight ahead pointing (SSA) task, showing a post-prism shift in both tasks towards the left. Numbers on the *x*-axis represent pointing error in degrees, with positive values indicating deviation to the right and negative values indicating deviation to the left. Note the similar size of the prism-induced shift for the two tasks in healthy controls.

**Fig. 4:**
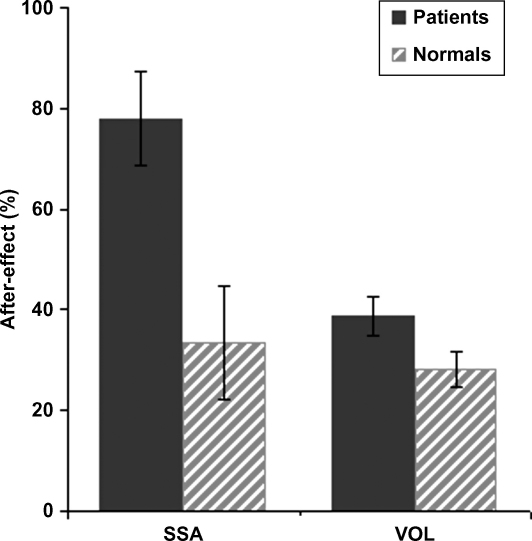
Size of aftereffect (post- minus pre-prism pointing error) for the group of neglect patients and separately for the group of age-matched healthy controls, for both the visual open-loop pointing (VOL) task and for the subjective straight ahead pointing (SSA) task. Numbers along the *y*-axis represent % of the total prism-induced optical displacement (e.g. a score of 50% would correspond to a 5° shift with the 10° prisms used). Note that our neglect patients show a pathologically large SSA shift due to prism exposure (see also Figs. 1 and 2), but a relatively normal VOL shift, as compared with the healthy controls.

The leftward aftereffect for SSA after prisms in our neglect patients (mean ∼8°) was significantly larger than the mean 3.35° (S.D. = 4.1°) SSA leftward aftereffect found for our age-matched healthy control group (*t*(12) = −2.99, *p* = 0.01). By contrast, the VOL aftereffect for the neglect group (mean 3.8°), did not differ significantly (*t*(12) = −1.85, n.s.) from that in the healthy controls (mean 2.82°, S.D. = 1.3°). The control group (see Fig. 3) shows a mild leftward deviation in the SSA task pre-adaptation (mean = −2.11°, S.D. = 2°), which differed substantially from the initial rightward bias in the neglect patients (*t*(12) = −6.57, *p* = 0.00003). For the VOL task prior to adaptation, healthy controls were overall quite accurate in pointing at the target (mean = 0.33°, S.D. = 1.5°), which did not differ significantly from the performance of patients (*t*(12) = −1.97, n.s.). Importantly, while after prism adaptation the mean pointing error for the control group shifted significantly towards the left for both tasks, this prism-induced leftward shift was equivalent for the SSA and VOL tasks (*t*(12) = 0.47, n.s.) quite unlike the much larger prism-induced shifts for SSA in the patients (see Fig. 4).

Thus our group of neglect patients showed a very large prism aftereffect on the SSA measure, with their substantial pathological bias towards the right in SSA prior to prisms (mean 8.76°) being effectively ‘corrected’ post-prisms. This very substantial shift in SSA, due to the prism intervention, was significantly larger than that demonstrated by our healthy controls (see Fig. 4). By contrast, our patients showed more modest (but still highly reliable) prism aftereffects on the VOL measure, but those fell well within the range of those demonstrated by normal controls (see Fig. 4). Finally, we note that whereas our patients clearly showed a much bigger prism aftereffect for the SSA than for the VOL measure, the normative data show no significant difference between these tasks in the healthy individuals tested here.

A referee queried if the difference in aftereffects for SSA and VOL in the patient group might perhaps reflect a faster decay rate of the VOL aftereffect compared to the SSA aftereffect (see Hatada et al., 2006). However, all of the post-prism pointing measures used here (for both SSA and VOL) were obtained quite rapidly (within a minute or so) of prism exposure. Moreover, when splitting our 10 repeated measures for each task into the first five versus the subsequent five, we found no effect of this (all *p* > 0.5, in both patients and controls). Finally, any normal difference in decay rates that may exist for the two aftereffects would presumably have applied to the healthy control group also, yet only the neglect group showed the substantial difference between SSA and VOL aftereffects that was observed in our patients.

We next consider whether any particular clinical features of our patients relate to the size of observed SSA or VOL aftereffects. The presence of hemianopia did not appear to have an impact, as no significant difference was found between patients with intact fields (*n* = 8) and patients with hemianopia (*n* = 5), in the size of the observed aftereffect (for SSA: *t*(11) = 1.19, *p* > 0.5; for VOL: *t*(11) = 0.36, *p* > 0.5). No reliable correlations were found between the SSA aftereffect and neglect severity on standard clinical tests prior to prisms (when measured by pre-prism cancellation, *ρ*(11) = 0.488, *p* = 0.09; or by line bisection performance, *ρ*(11) = −0.294, *p* = 0.33); nor with current age; nor with time post-stroke (*p* > 0.2 for both); likewise for the VOL aftereffects also (for all those correlation results, *p* > 0.2).

### 3.2 Prism effect on cancellation; and any relation of this to SSA or VOL prism aftereffects

**Fig. 5:**
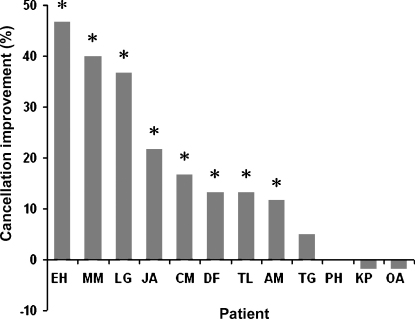
Percentage of improvement in Mesulam cancellation for each individual patient, for post- minus pre-prism scores. Patients are arranged along the *x*-axis in terms of size of improvement. Asterisks (*) indicate an individually significant improvement.

Although our main focus was on contrasting the SSA versus VOL measure, for completeness we had also implemented the Mesulam cancellation test both pre- and post-prisms in our patient group. Since patient CO was already close to ceiling on this cancellation task even before the prism procedure (cancelling 95% of the target items at that point, although she had shown substantial neglect on cancellation closer to the original onset of her injury) we excluded her cancellation data from further consideration. For the remaining 12 patients, eight showed some individually significant improvement in cancellation after prism adaptation (JA cancelled 46/60 targets pre and 59/60 post, *p* < 0.001; EH cancelled 28 pre and 56 post, *p* < 0.001; AM cancelled 49 pre and 56 post, *p* < 0.05; CM cancelled 14 pre and 24 post, *p* = 0.05; LG cancelled 33 pre and 55 post, *p* < 0.001; MM cancelled 16 pre and 40 post, *p* < 0.001; TL cancelled 46 pre and 54 post, *p* < 0.05). There was a substantial trend towards improvement in the case of DF (who cancelled 44 of the targets pre and 52 post, *p* = 0.068), which became significant when considering only the left side of the cancellation page (cancelling 14/30 of the targets on that side pre and 22/30 post, *p* = 0.035). By contrast, KP, OA, TG and PH showed no improvement in cancellation (KP cancelled 8 of the targets pre and 7 post, *p* = 0.78; OA cancelled 33 of the targets pre and 32 post, *p* = 0.85; TG cancelled 7 of the targets pre and 10 post, *p* = 0.43; PH cancelled 10 of the targets pre and 10 post). See Fig. 5 for a summary of these individual patient results in cancellation. When considering those patients who did show prism-induced improvement in cancellation (*n* = 8) as a separate subgroup from those who did not (*n* = 4), we found that the prism-induced SSA aftereffect was larger in the former versus the latter group (*t*(10) = 3.9, *p* = 0.003), whereas the VOL aftereffect did not differ (*t*(10) = −0.9, *p* = 0.3). This hints at a possible relationship between the SSA aftereffect and improvements in neglect on cancellation after prisms, as explored further below.

**Fig. 6:**
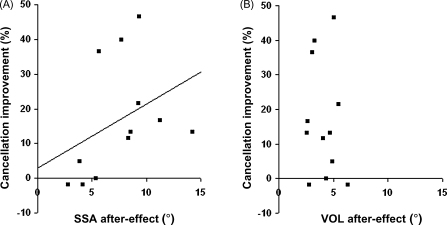
(A) SSA or (B) VOL prism aftereffects (i.e. leftward shifts for post- minus pre-prism pointing error, in degrees of visual angle along the *x*-axis), each plotted against cancellation improvement after prism adaptation along the *y*-axis (in percentage change), with each point corresponding to one patient from the 12 considered. A significant correlation was found between cancellation improvement and the prism-induced SSA shift (see (A), with regression line shown); but there was no such correlation between cancellation improvement and the VOL shift, as apparent in (B).

The extent of improvement in cancellation post-prism was not found to correlate overall with the initial deficit in this task prior to prisms (on number of leftward omissions; *ρ*(10) = 0.25, *p* > 0.05); nor with current age (*ρ*(10) = 0.15, *p* > 0.05); nor with time post-stroke (*ρ*(10) = −0.33, *p* > 0.05); nor to relate to the presence/absence of hemianopia (*t*(11) = 1.38, *p* > 0.05). On the other hand, there was a significant correlation between improvement in cancellation post- versus pre-prism, with the corresponding individual shift in SSA (*ρ*(10) = 0.625, *p* = 0.03); see Fig. 6A. This contrasted with no such relationship between cancellation improvements and the prism-induced VOL shift (*ρ*(10) = −0.063, *p* = 0.845); see Fig. 6B.

### 3.3 Regions of selective lesion overlap in patients failing to show a prism benefit versus those showing amelioration of neglect by prisms

To provide an initial exploration of any possible differences in lesion anatomy, patients were sorted into two groups (an ‘improved’ and a ‘non-improved’ patient subgroup) according to whether or not as individuals they showed a significant improvement in their neglect for *cancellation* after the prism intervention, compared with before (see above). While any anatomical differences would require confirmation in a study with larger patient numbers, we present the lesion information here for completeness and for comparison with future studies.

Lesion information was available from clinical scans for 11 of the 12 neglect patients who completed the cancellation task (we were unable to obtain lesion data for patient TL). MRI scans were available for seven patients and CT scans for five. The extent and location of lesions were defined and visualized using the MRIcro software package (Rorden & Brett, 2000; www.mricro.com) by one of the authors (MH, a clinical neurologist), when blind as to the behavioural performance of each patient. Lesions were drawn manually on a T1-weighted template MRI scan from the Montreal Neurological Institute (www.bic.mni.mcgill.ca/cgi/icbm_view), thus in ‘MNI’ space, on 12 axial slices corresponding to Z-coordinates −16, −11, −6, −3, 3, 13, 16, 20, 24, 30, 36 and 48 mm, by using the identical or closest matching transverse slices for each individual. Combining all slices produced a 3D lesion ROI for each patient.

**Fig. 7:**
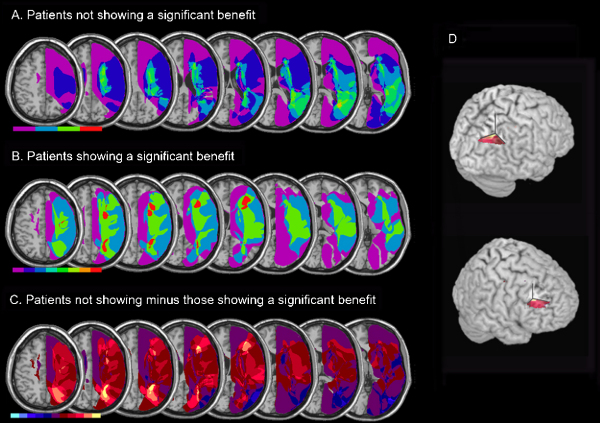
Lesion contrasts for non-improved vs. improved patients, in terms of the impact of prism exposure on neglect in cancellation. (A) Non-improved patients (*n* = 4). Overlap map showing the degree of involvement for each voxel in the lesions of the non-improved neglect group, normalized to the MNI template. The map is presented as axial renderings on the MNI ‘representative’ brain, in descending steps. Eight axial slices are shown that correspond to Z-coordinates 48, 36, 30, 24, 20, 16, 13 and 3 of the MNI space. The range of the colour scale derives from the absolute number of patient lesions. (B) Overlap map for the prism-improved neglect patients (*n* = 7). (C) Non-improved minus improved patients. Contrast map showing the relative involvement (bins of 16.67%, apart from the purple shading which represents −16.67% through to +16.67%) of each voxel in the lesions of the non-improved patient group minus the improved patient group. The colour scale covers a range of voxel involvement in the two lesion groups, from involvement in the non-improved neglect group only (light yellow) to involvement in the improved neglect group only (light blue). (D) Rendered views of the regions of lesion overlap differentially involved in neglect patients who did not show improvement on cancellation after prisms, minus those who did (approximate centre of clusters in MNI coordinates: 24, −59, 36; 27, 28, 24).

Via MRIcro software we generated voxel-based lesion-overlap maps separately for those subgroups of patients who did (KP, OA, TG and PH) or did not (EH, JA, AM, DF, CM, LG and MM) show a cancellation improvement due to prisms; see top two rows of Fig. 7. We then subtracted (see Karnath, Ferber, & Himmelbach, 2001; Mort et al., 2003) these to identify any regions particularly involved in those patients who did not show a prism benefit, as compared with those who did (Fig. 7C and D). This highlighted involvement of the right intraparietal region and white matter deep to inferior parietal lobe (approximate centres of clusters in MNI coordinates: 24, −59, 36; 25, −51, 30). Moreover, a much smaller region of overlap was found in white matter of the right middle frontal gyrus (approximate centre of clusters in MNI coordinates: 27, 28, 24; 28, 37, 20). These regions were damaged in all four patients failing to show an improvement after prism adaptation, but in none of the seven patients who did show an improvement. Mean lesion volume between cancellation improvers and non-improvers did not differ reliably (*U* = 11.000, *N*_1_ = 7, *N*_2_ = 4, *p* = 0.571). Moreover we found no correlation between lesion size and improvement in cancellation post-prisms (*ρ*(9) = −0.183, *p* = 0.6).

## 4 Discussion

The present study investigated possible differences between two frequently used but substantively different methods of assessing prism adaptation in neglect patients, namely the subjective straight ahead (SSA) and visual open-loop (VOL) pointing tasks, also commonly used in the normal literature to assess prism adaptation. In normals, both these measures typically reveal prism aftereffects in the form of shifts of pointing in the opposite direction to the exposed prismatic optical deviation. Prior to conducting the present study we had several reasons to suspect that one of these aftereffect measures (SSA) may produce pathologically large aftereffects in neglect patients, but not the other (VOL). First, some of the existing results on prism interventions in the neglect literature suggest indirectly (when considered collectively, as in our new meta-analysis of 14 previously published neglect studies using *either* SSA *or* VOL to measure prism aftereffects, but never both in the same patients, unlike here; see Section 1) that the SSA task might reveal pathologically large aftereffects in neglect patients following exposure to rightward deviating prisms, whereas the VOL task may not. Second, theoretical considerations concerning neglect led us to hypothesize that the SSA task may often reveal apparently larger prism adaptation in neglect patients, than the VOL measure and than in normals, because the SSA task in particular may tap into a characteristic abnormality in neglect (namely pathological deviation of the subjective straight-ahead, cf. Karnath (1994, 1997)) that might benefit from appropriate prism therapy. This may not apply to the VOL task, which might be somewhat closer to providing a ‘pure’ measure of prism adaptation per se in neglect.

With these considerations in mind, we examined aftereffects of a brief exposure to 10° rightward optical prisms, on *both* the SSA *and* the VOL task (implemented after a single session of prism exposure so that the prism manipulation itself was identical for both tasks), within the *same* sample of 13 consecutive right-hemisphere patients with left neglect, and compared them to a sample of 13 age-matched controls. We deliberately made the SSA and VOL tasks as similar as possible (e.g. both required the same number of responses, and the objectively ‘correct’ response was also identical for both tasks). For completeness, we also implemented a standard clinical test for neglect (cancellation) both pre- and post-prisms, to assess any prism impact on neglect as conventionally defined, and any relation of such improvements to prism-induced improvements in SSA or VOL.

We found that while reliable prism aftereffects were obtained in the group of neglect patents (in all individuals) for both the SSA and VOL measures, the aftereffect for SSA was much larger (mean 7.8° leftward) than the VOL aftereffect (mean 3.9° leftward), with SSA responses post-prisms becoming close to the objective midline for the first time; see Figs. 1 and 2. The new results for our patient group as a whole are similar to the mean aftereffects reported in previous studies of neglect that used a similar methodology (10° prisms), but which had applied only *one or other* of the two aftereffect measures in neglect patients (see our new meta-analysis in the Section 1). We thus confirmed the pattern that we had predicted, of significantly larger prism aftereffects on SSA than on VOL in neglect patients, now within the very same group of patients, after an identical prism intervention.

We were also able to confirm that this pattern of results for neglect patients is pathological. A direct comparison of the patient data with an age-matched healthy control group revealed that while the present SSA shifts were pathologically large in the neglect patients, the VOL neglect results clearly fell within the normative range. Moreover, the SSA and VOL aftereffects did not differ in normals with the paradigm used here. Thus, the SSA measure reveals pathologically large prism aftereffects in patients with left neglect (after exposure to rightward prismatic deviation); while VOL prism aftereffects are perfectly normal in the very same patients (see Fig. 4).

One explanation for this could relate to the fact that distortions in the subjective straight ahead (as indexed in the SSA task) have been claimed to provide one contributory component to neglect (e.g. Jeannerod & Biguer, 1987; Karnath, 1994, 1997; Karnath & Fetter, 1995). Indeed, as shown here and in numerous other studies (e.g. Chokron & Bartolomeo, 2000; Heilman et al., 1983; Pisella et al., 2002; Richard et al., 2004; Rossetti et al., 1998; Vallar et al., 1997), when neglect patients are asked to point straight ahead in the dark or blindfolded, they typically deviate substantially to the right of their objective body midline. This pathological bias in SSA has been suggested by Karnath and co-workers to reflect a pathological shift of egocentric reference frame(s), which might arguably play some role in other neglect manifestations, including ipsilesional biases in visual or tactile exploration (e.g. see Karnath, 1994, 1997; see also Jeannerod & Biguer, 1987). Some studies have challenged Karnath's initial proposal that SSA distortion may be the ‘primary’ cause of neglect, by showing that SSA abnormalities may not be exclusive to neglect, may not be present in all neglect patients and may not always correlate with performance on other neglect tests (Bartolomeo & Chokron, 1999; Chokron & Bartolomeo, 1997; Chokron et al., 2002; Farne, Ponti, & Ladavas, 1998; though see Richard et al., 2004; Schindler et al., 2002). The fact remains, however, that the rightward shift in SSA is a well established and frequently found component of the neglect syndrome, although of course it seems unlikely to account for *all* other neglect manifestations, given the emerging consensus that neglect is a multi-component syndrome.

Prism effects upon the distorted SSA might thus in principle be considered as one further manifestation of the beneficial impact of prisms upon the neglect syndrome, rather than as providing a ‘neglect-free’ measure of prism adaptation per se (that might be measured more purely by VOL in neglect patients). If so, this may explain the ‘extraordinary’ (i.e. pathologically large; see also Redding & Wallace (2006) and Rossetti et al. (1998)) effect of prisms on the SSA task in the neglect patients; and also the lack of such exceptional prism effects for the VOL task, in which neglect patients perform similarly to normals. Interestingly, improvement for the SSA in neglect has now also been reported following other interventions, such as plantar stimulation (Richard, Rousseaux, & Honore, 2001), neck muscle vibration or caloric vestibular stimulation (Karnath, 1994) and manipulations of spatial attention (Richard, Rousseaux, & Honore, 2005). Moreover, such an interpretation might also accord with another aspect of the findings here, namely that aftereffects for SSA (but not for VOL) correlated with the extent of post-prism improvement in cancellation (see Fig. 6A).

Further recent evidence on prism effects in neglect has been taken to suggest that prism interventions may enhance strategic or ‘endogenous’ aspects of orienting towards the affected side in neglect patients, more than for automatic or ‘exogenous’ aspects of such orienting (Nijboer, McIntosh, Nys, Dijkerman, & Milner, 2007; but see also Striemer & Danckert, 2007). From this perspective, one might speculate that the present pathologically large effect of the prism intervention on SSA may reflect some strategic or endogenous component, whereas the relatively normal VOL effect may reflect more automatic consequences of exposure to prisms and the sensorimotor recalibration that this induces.

There are now numerous reports demonstrating some beneficial effects of prism adaptation on various aspects of the neglect syndrome (see Section 1), as also found for the SSA and cancellation measures here, but a key remaining question concerns the causal mechanisms behind such amelioration. A number of recent studies sought to investigate how the duration (Frassinetti et al., 2002; Farne et al., 2002; Pisella et al., 2002) and/or size (Serino et al., 2006) of the ‘conventional’ prism aftereffect may relate to the duration or size of improvements in neglect. Some studies reported no clear links between the conventional aftereffect (but this was usually measured by VOL, rather than by SSA), and neglect improvement for clinical tests (e.g. Girardi, McIntosh, Michel, Vallar, & Rossetti, 2004; Frassinetti et al., 2002; Pisella et al., 2002; and in particular, Serino et al., 2006). But some other work indicates that a relationship between these can sometimes arise (Farne et al., 2002). The present study highlights a key factor that may need to be considered in any future work on this issue, indicating that the method of assessing prism adaptation may be crucial. Here we found that neglect improvement may relate more to the SSA than to the VOL aftereffect, and that only the former is pathologically large.

The present study also allowed us to perform an exploratory assessment of the potential anatomical correlates of neglect improvement following prism therapy, via lesion subtraction. We compared the lesions of those four cases who did not show prism-induced cancellation improvement, against the larger group who did (see Fig. 7). This indicated that candidate brain regions potentially associated with *lack* of neglect improvement following prism therapy may be the right intraparietal region plus white matter deep to inferior parietal lobe, as well as white matter in the right middle frontal gyrus. Lesion volume per se was not significantly related to the behavioural outcome after prism intervention. These anatomical results should be considered preliminary, as a larger number of patients is needed to confirm and refine them. Nevertheless, the highlighted regions (see Fig. 7D) seem of potential interest in several respects. First, while relatively little is currently known about the exact neural basis of prism adaptation in the normal brain, the limited evidence (see Clower et al., 1996; Newport, Brown, Husain, Mort, & Jackson, 2006; Newport & Jackson, 2006; Pisella et al., 2004, 2005; Pisella, Rode, Farne, Tilikete, & Rossetti, 2006; Redding, Rossetti, & Wallace, 2005; Weiner, Hallett, & Funkenstein, 1983) points to a cerebellar-posterior parietal (PPC) network, with PPC possibly contributing to more strategic or ‘cognitive’ aspects of adaptation, and the cerebellum to plastic recalibration between visual, proprioceptive and motor correspondences. It is conceivable that damage in PPC, and/or the white-matter fibres connecting the PPC to the cerebellum, could thus disrupt visuo-motor adaptation, leading to reduced effects in some neglect patients. But it may be important to note that here all our patients (including those few who demonstrated no post-prism improvement in cancellation) showed some reliable prism *aftereffects* (both in VOL and SSA) and therefore evidence for at least some adaptation to prisms. Thus the lack of beneficial effects on neglect in these patients cannot be attributed to an absence of prism adaptation per se. This further underlines that the presence of a significant prism aftereffect (e.g. on VOL) does not guarantee amelioration of the patient's neglect symptoms; and that damage to the right intraparietal (and underlying) region highlighted here might disrupt neglect improvement due to prisms, but not prism adaptation per se.

Other recent findings also suggest that the right infero-posterior parietal lobe, generally agreed to be implicated in multisensory integration and sensorimotor translation (Andersen, Snyder, Bradley, & Xing, 1997; Driver & Spence, 2000; Macaluso & Driver, 2005; Xing & Andersen, 2000), may when spared be an important site for short- and long-term beneficial effects due to prism therapy in visuo-spatial neglect. Luaute, Michel, et al. (2006) recently used PET illustrating potential involvement of the right posterior parietal lobe in beneficial effects of prism adaptation for neglect patients. They also noted that among their patients those who showed only marginal improvement following prism adaptation (two of the five they tested) were also the only ones to have lesions involving right infero-posterior parietal cortex. The involvement of infero-posterior parietal cortex in neglect improvement, both during natural recovery (Corbetta, Kincade, Lewis, Snyder, & Sapir, 2005) and following rehabilitative interventions (prism adaptation, as here and in Luaute, Michel, et al. (2006); or alertness retraining, as in Thimm, Fink, Kust, Karbe, and Sturm (2006)) suggests that there may be a fairly general role for this region in neglect improvement, when intact.

In conclusion, the present study highlights the need for future work on relations between prism aftereffects and possible mechanisms underlying prism-related amelioration of neglect, to carefully consider the tasks employed. We found clearly that SSA and VOL tasks show different patterns of prism aftereffects in neglect patients that do not cross-correlate. The SSA task reveals pathologically large prism aftereffects in right-hemisphere left neglect patients as compared to normal subjects, whereas the same patients appear entirely normal on the VOL measure of prism aftereffects. We suggest that this could arise because SSA may not provide a strictly ‘pure’ measure of prism adaptation in neglect patients, as the post-prism shift in SSA may also reflect one aspect of neglect improvement. Indeed, we found that the size of the SSA shift correlated with improvements in another aspect of neglect following prism exposure, namely cancellation. Finally, preliminary lesion information implicated the right intraparietal region (and underlying white matter), as well as white matter in the right middle frontal gyrus, as regions that when preserved may play a role in neglect improvement after prism therapy, and which when damaged might explain why not all patients show such improvements. Further work in a larger group of patients is required to confirm and refine anatomical considerations. But the present sample of 13 neglect patients already shows clearly that prism aftereffects are pathological for the SSA measure, but not for the VOL measure; and that the SSA shift (but not the VOL aftereffect) can relate to improvements in other manifestations of neglect. SSA and VOL should thus no longer be considered interchangeably when assessing prism adaptation in neglect.

## Figures and Tables

**Table 1:** Summary of individual patient details and test scores

Patient	Sex	Age	Cancellation	Line bis. (%)	Chimeric objects (%)	Post-stroke	Hemianopia	Lesion site and pathology
KP	M	51	L:0; R:8	78	n/a	16	Yes	Right parietal-temporal lobectomy following SDH and bleed
DF	M	72	L:16; R:30	−8	59	174	No	Right MCA infarct involving basal ganglia and white matter
EH	F	59	L:0; R:28	65	0	16	Yes	Right SAH in the MCA territory with extension into the sylvian fissure
JA	M	69	L:16; R:30	2	n/a	1	No	Right MCA infarct involving the parietal and extending into the occipital lobe
OA	M	41	L:3; R:30	1.4	100	23	No	Right MCA infarct involving the inferior parietal and frontal lobes
PH	M	51	L:0; R:10	9	11	13	Yes	Right intracerebral bleed with subarachnoid extension
AM	M	67	L:23; R:29	74	35	1	No	Right MCA infarct involving temporal and parietal lobes
CO	F	57	L:25; R:30	23	39	4	No	Right MCA infarct involving frontal lobe, basal ganglia and insular cortex
CM	F	64	L:0; R:14	61	n/a	2	No	Right MCA infarct, including frontal lobe white matter and basal ganglia
LG	F	23	L:4; R:29	39	0	5	Yes	Right MCA infarct
MM	F	60	L:13; R:30	25	25	3	Yes	Right parieto-occipital PCA/MCA ‘watershed’ infarct
TG	F	77	L:13; R:30	78	n/a	2	No	Right ACA & MCA infarct
TL	M	58	L:16; R:30	−7	94	4	No	Right ACA infarct

*Notes*. *Cancellation score*: total number of targets cancelled on the left and the right side of the page in the Mesulam shape cancellation task, out of 30 per half-page; *Line bis*.: mean percentage of deviation towards the right for five 18 cm lines; *Chimeric objects*: percentage of chimeric objects correctly identified (on both the left and the right side; out of a total of 46 each); *Post-stroke*: time post-stroke in months at the time of the experiment; *n*/*a*: indicates non-available data, due to clinical constraints; MCA: middle cerebral artery; ACA: anterior cerebral artery; SAH: subarachnoid haemorrhage.
